# Microbial Community and Metabolite Dynamics During Soy Sauce *Koji* Making

**DOI:** 10.3389/fmicb.2022.841529

**Published:** 2022-02-25

**Authors:** Guiliang Tan, Min Hu, Xiangli Li, Xueyan Li, Ziqiang Pan, Mei Li, Lin Li, Yi Wang, Ziyi Zheng

**Affiliations:** ^1^School of Material Science and Food Engineering, Zhongshan Institute, University of Electronic Science and Technology of China, Zhongshan, China; ^2^School of Environmental and Safety Engineering, Changzhou University, Changzhou, China; ^3^School of Health Industry, Zhongshan Torch Vocational and Technical College, Zhongshan, China

**Keywords:** *koji* making, metagenome, microbial community structure, metabolites, functional potential

## Abstract

Koji making is a pre-fermentation stage in soy sauce manufacturing that impacts final product quality. Previous studies have provided valuable insights into the microbial species present in *koji*. However, changes in microbial community functional potential during *koji*-making are not well-known, nor are the associations among microbial populations and flavoring characteristics. In the present study, we investigated the succession of microbial communities, microbial community functional potential, metabolite profiles, and associations among microbial community members/functions with metabolites during *koji* making using shotgun metagenomic and metabolomic analyses. *Firmicutes*, *Proteobacteria*, and *Ascomycota* were identified as the most abundant microbial phyla in early *koji* making (0–12 h). *Aspergillus* (fungi) and *Weissella* (bacteria) exhibited marked abundance increases (0.98–38.45% and 0.31–30.41%, respectively) after 48 h of fermentation. Metabolite analysis revealed that aspartic acid, lysine, methyl acetate, isovaleraldehyde, and isoamyl alcohol concentrations increased ∼7-, 9-, 5-, 49-, and 10-fold after 48 h of fermentation. Metagenomic profiling demonstrated that *koji* communities were dominated by genes related to carbohydrate metabolism and amino acid metabolism, but functional profiles exhibited marked shifts after 24 h of fermentation. The abundances of genes within the categories of carbohydrate and amino acid metabolism all increased during *koji* making, except for pyruvate metabolism, glycolysis/gluconeogenesis, and the citrate cycle. Correlational analyses indicated that *Aspergillus*, *Lactococcus*, *Enterococcus*, *Corynebacterium*, and *Kocuria* abundances were positively correlated with 15 amino acid concentrations (all *p* < 0.05), while *Weissella* abundances were positively correlated with concentrations of volatile flavor compounds, including eight amino acids, phenylacetaldehyde, acetic acid, 2,3-butanediol, ethyl acetate, and ethanol (*p* < 0.05). These results provide valuable information for understanding the microbial-associated mechanisms of flavor formation during *koji* making.

## Introduction

Soy sauce is a traditional fermented soybean product that originated in China over 2,500 years ago (Feng et al., 2013). Soy sauce has distinctive, characteristic tastes and aromas and is consumed as an essential condiment in China and other Asian countries (Wei C. L. et al., 2013). Soy sauce production involves two fermentation stages: *koji* making and *moromi* fermentation. *Koji* is a solid-state fermentation of steamed whole or defatted soybeans and wheat flour using filamentous fungi (e.g., *Aspergillus oryzae* or Aspergillus *sojae*), with an incubation time from 26 h to 7 days (Wei C. L. et al., 2013). *Moromi* is a mixture of *koji* and a brine solution containing 18–22% NaCl that is allowed to spontaneously ferment for 3–6 months (Devanthi and Gkatzionis, 2019).

*Koji* making is the first step in soy sauce brewing and is an extremely important step in determining the quality of final products (Yan et al., 2013; Ding et al., 2019; Zhao et al., 2020). For example, most of the volatile compounds present in initial *moromi* fermentations might develop in the *koji*-making stage (Gao et al., 2010; Feng et al., 2013). During *koji* making, molds (e.g., *Aspergillus*) produce proteolytic enzymes that hydrolyze proteins into peptides and amino acids, in addition to amylases that also convert starches into simple sugars (Devanthi and Gkatzionis, 2019). Many metabolites have been identified in *koji* samples including volatile flavor compounds (VFCs), fatty acids, and lipids. Among these, aldehydes and alcohols are the major volatile compounds (Feng et al., 2013), and triacylglycerols are the most abundant fatty acids (Feng et al., 2014). In addition, *koji* making also provides enzymes (such as proteases and amylases) for the hydrolysis of raw materials in the subsequent brine fermentation, which then affects soy sauce chemical compositions, colors, and flavors (Wicklow et al., 2007).

Microorganisms play essential roles in flavor development during soy sauce fermentation (Devanthi and Gkatzionis, 2019; Mannaa et al., 2020). In particular, the bacterial genera *Weissella*, *Staphylococcus*, *Tetragenococcus*, and *Bacillus*; in addition to the fungal genera *Aspergillus*, *Zygosaccharomyces*, *Candida*, and *Debaryomyces* predominate throughout the *moromi* fermentation stage (Wei Q. Z. et al., 2013; Sulaiman et al., 2014; Han et al., 2020). However, few studies have evaluated the microbial community compositions and their contributions to flavor development during *koji* making. The non-sterile environment of *koji* making leads to contributions to flavoring from diverse microorganisms including *Weissella*, *Staphylococcus*, *Lactobacillus*, *Streptococcus*, *Enterococcus*, *Kurthia*, and *Klebsiella* (Tanaka et al., 2012; Wei Q. Z. et al., 2013; Yan et al., 2013). Among these, *Weissella* and *Staphylococcus* have been observed as the predominant bacterial genera in *koji*, while *Aspergillus* is the dominant fungal genus. In addition, less abundant fungal genera are present including *Candida*, *Wickerhamomyces*, *Pichia*, *Geotrichum*, and *Trichosporon* (Tanaka et al., 2012; Wei Q. Z. et al., 2013; Yan et al., 2013). Previous studies have provided valuable insights into the microbial communities present in *koji*, but changes in the functional potentials of microbial communities during *koji* making are not well known and correlations between microflora and metabolites are underexplored. The composition of microorganisms in soy sauce production (and in *moromi* fermentation) has been investigated primarily by 16S rRNA or ITS gene amplicon sequencing (Liang et al., 2019; Guo et al., 2020; Han et al., 2020; Liu et al., 2021; Qi et al., 2021), in addition to shotgun metagenomics (Sulaiman et al., 2014; Kim et al., 2021). A previous metagenomic study has shown that as fermentation progressed, microbial diversity decreased in the mid to late stages of *moromi* fermentation (Sulaiman et al., 2014; Kim et al., 2021), while inference of functional potential suggested characteristic profiles involved with heterotrophic fermentation of proteins and carbohydrates (Sulaiman et al., 2014). These studies have expanded our understanding of microbial community structure and functions in soy sauce *moromi* fermentation. However, the capacities of microbial communities for flavor generation during *koji* making requires further investigation.

In the present study, we investigated the microbial succession, community functional potential, and metabolite profiles present during *koji* making, while also evaluating correlations among microbial community composition/functions with metabolites using metagenomic and metabolomic analyses. These results provide a better understanding of the roles of microorganisms in *koji* making and flavor generation that can then be used to improve soy sauce product qualities.

## Materials and Methods

### The *Koji* Making Process and Sample Collection

*Koji* samples from high-salt liquid-state fermentations of soy sauce were collected from the Pearl River Bridge Biotechnology Co., Ltd (Zhongshan, Guangdong, China), which is one of the most famous food manufacturing companies in China. Prior to *koji* making, whole soybeans were steam-cooked and the steamed soybeans were mixed with wheat flour at a ratio of 3:1 (w/w), followed by cooling to 30°C and inoculation with 0.03% *A. oryzae* strain 3.042 as a spore starter (Feng et al., 2013). Subsequently, mixtures were fermented in a vessel (length: 8 m × width: 4 m × depth: 50 cm) at 28–35°C in a *koji* incubation room. Relative humidity was maintained at about 95% during *koji* making. The overall process of *koji* making is illustrated in Supplementary Figure 1. To investigate the succession of microbial communities and metabolites during the process, *koji* samples were periodically taken at 0–48 h. At each sampling time, 50 g of *koji* samples were randomly collected from three vessels, placed in 50 mL centrifuge tubes (Corning CentriStar, NY, United States), immediately transported on ice to the laboratory, and then stored at −20°C until subsequent DNA extraction and chemical analysis.

### Determination of Chemical Characteristics

To measure sample pH, 10 g of *koji* sample was mixed with 100 mL of distilled water, and then centrifuged (9,000 × *g*, 10 min) at room temperature. The distilled water was heated to boiling and then cooled to room temperature before use. The supernatant pH was measured directly with a PB-10 pH meter (Sartorius, Göttingen, Germany). Free amino acid (FAA) contents were detected using ultra-HPLC tandem MS (UPLC-MS/MS; model 1290/6460; Agilent Technologies, Santa Clara, CA, United States). Briefly, samples were extracted with distilled water (pH 3.0) and the extracted solutions were purified using hexane (Merck, Germany). The FAAs were then separated on an ACQUITY UPLC BEH HILIC (2.1 × 100 mm, 1.7 μm; Waters Corp.) using ammonium formate-acetonitrile/ammonium formate-H_2_O (pH 3.0) as the mobile phase and detected with MS/MS using multiple reaction monitoring modes (Tan et al., 2020).

Volatile flavor compounds were analyzed as described previously (Feng et al., 2013), with minor modifications. Briefly, *koji* samples (2.5 g) were mixed with 0.5 g of NaCl and 20 μL of 2-methyl-3-heptanone (2 mg/L in methanol) as an internal standard in 15 mL amber SPME vials, followed by equilibration for 15 min with a thermostatic water bath at 55°C. VFCs were then extracted with SPME fiber (CAR/PDMS, 75 μm; Supelco Co., Bellefonte, PA, United States) at 55°C for 30 min. VFCs were analyzed using a GC-MS system (model 6890N/5975; Agilent Technologies, Santa Clara, CA, United States). The oven temperature gradient for GC-MS started at 33°C (2 min) and then increased at 5°C/min to 70°C, followed by increases at 10°C^/^min to 250°C. GC-MS settings included an injector temperature of 250°C and a run time of 30 min. Compounds were identified by comparison with mass spectral data from the NIST 14 mass spectra database. All extractions were conducted in triplicate. The concentrations of each volatile component in *koji* samples were quantified by comparing their peak areas to those of internal standard compounds on the total GC-MS ion chromatograms. All of the quantitative data represent mean values for triplicate measurements.

### Community DNA Extraction and Metagenome Sequencing

Total genomic DNA from *koji* samples (0.5 g) was extracted using an EZNA™ Mag-Bind Food DNA kit (Omega Bio-Tek, Inc., Norcross, GA, United States) according to the manufacturer’s instructions. Triplicate samples of extracted DNA from the same sample time were combined for downstream metagenomic sequencing. DNAs were then sheared into 300 bp fragments and sequenced on the Illumina HiSeqX-Ten platform (Illumina Inc., San Diego, CA, United States) to generate 2 × 150-bp paired-end sequencing reads. Sequencing was performed at the Novogene Bioinformatics Technology facility (Beijing, China).

### Metagenomic Bioinformatics

Sequencing adapters were removed from reads that were then trimmed with Trimmomatic v 0.30 using a quality cutoff of 30, a sliding window of 6 bp, and a minimum length cutoff of 45 bp (Bolger et al., 2014). High quality-filtered reads were pooled and assembled with the IDBA-UD 1.1.1 assembler (Peng et al., 2012) to obtain contigs using a minimum *k* value of 60; a maximum *k* value of 120; incremented *k*-mer sizes of 10 for each iteration; a minimum multiplicity of 5 for filtering *k*-mers when building the graph; a seed *k*-mer size of 5 for alignment; and a minimum contig length of 1,000; while all other parameters were set to default values. Contigs shorter than 500 bp were excluded. Genes of the assembled contigs were predicted using the MetaGeneMark software program (Zhu et al., 2010) and genes shorter than 300 nt were removed. The CD-HIT software program (Fu et al., 2012) was used to remove redundant genes at a threshold of 95% nucleotide identity and ≥90% coverage, with the longest sequence in each gene cluster used for downstream analyses. Non-redundant gene sets were aligned to the National Center for Biotechnology Information (NCBI)-nr database using the DIAMOND aligner (Buchfink et al., 2015) and an e-value threshold of <e^–5^, followed by taxonomic profiling of genes with MEtaGenome ANalyzer (MEGAN) (Huson et al., 2007). Plant-associated genes were removed from the dataset to reduce the effects of plant genome contamination on the results. Thus, only bacterial- and fungal-affiliated genes were retained for downstream analysis. The filtered reads were subsequently mapped back to the microbial genes using Bowtie2 with default parameters, and the tags per million (TPM) values were calculated for each gene and their corresponding taxa to reduce the effects of sequencing depth and gene length on gene abundances. The taxonomic composition of *koji* metagenomes were then calculated by summing the TPM values for each lineage. To determine the relative abundances of genes in each sample, filtered reads were mapped back to the assembled microbial genes using Bowtie2 with default parameters. The functional profiles of the microbial communities were obtained by comparing the non-redundant microbial gene set against the Kyoto Encyclopedia of Genes and Genomes (KEGG) database (Kanehisa and Goto, 2000) using KOBAS 3.0 with a threshold of e^–5^ (Xie et al., 2011). Gene abundances were normalized based on the Z scores of the TPM values for each stage.

### Statistical Analyses

All statistical analyses were conducted using the SPSS 18.0 Software package (SPSS Inc., Chicago, IL, United States). Differences in datasets were evaluated by conducting one-way ANOVA tests followed by least significant difference (LSD) tests. Differences were considered significant at *p* < 0.05. The unweighted pair group method with arithmetic mean analysis (UPGMA) hierarchical clustering was used to assess community compositional similarities based on Bray-Curtis distances with microbial abundance data within the R vegan package v.2.5-7 (Oksanen et al., 2019). Correlations among microbial compositional characteristics and chemical properties were estimated by Spearman’s correlation coefficients, and strong correlations were identified by values of | ρ| > 0.7 and a *p* < 0.05. Heatmap visualization was constructed in the R environment with the “vegan” package.

### Sequence Accession

The metagenomic sequence data produced here are publicly available in the NCBI database under the BioProject accession PRJNA730347.

## Results and Discussion

### Changes in Chemical Characteristics During *Koji* Making

The pH slightly decreased during *koji* making from an initial value of 6.73 ± 0.06 to 6.33 ± 0.03 (Supplementary Figure 2). The contents of the 16 investigated amino acids all exhibited increased abundances with time (*p* < 0.05). In particular, arginine, aspartic acid, lysine, and phenylalanine were the predominant amino acid species at the end of fermentation (48 h), reaching concentrations of 12.44 ± 0.18 g/kg, 10.42 ± 0.03 g/kg, 10.10 ± 0.03 g/kg, and 8.11 ± 0.32 g/kg, respectively (Table 1). These amino acids might derive from the activity of the starter *A. oryzae* culture that hydrolyzes proteins into peptides and amino acids. In addition, a total of 66 VFCs were identified during *koji* making, including 12 esters, 10 alcohols, 10 aldehydes, 6 ketones, 4 acids, and 24 other compounds (Supplementary Table 1). The predominant volatile groups in the 48 h samples were aldehydes (31.22 ± 5.66%), alcohols (17.43 ± 1.31%), and esters (18.92 ± 3.90%), consistent with a previous study wherein the predominant volatile groups in *koji* samples were aldehydes and alcohols (Feng et al., 2013). The most abundant VFCs in the 48 h sample included isovaleraldehyde (with an average value of 939.75 μg/kg), 2,2,4,6,6-pentamethylheptane (470.53 μg/kg), methyl acetate (422.03 μg/kg), 1-octen-3-ol (385.46 μg/kg), and isoamyl alcohol (276.65 μg/kg) that exhibited approximately 49-, 3-, 5-, 1-, and 10-fold higher concentrations, respectively, compared to the initial 0 h sample (Supplementary Table 1). These results were consistent with those of a previous study wherein 3-methyl-butanal (isovaleraldehyde), 1-octen-3-ol, benzeneacetaldehyde, (E)-2-octenal, and benzaldehyde were the most abundant compounds (Feng et al., 2013). Among these, isovaleraldehyde is a branched short-chain aldehyde and is mainly produced from branched-chain amino acids *via* the Ehrlich pathway involving various fungal enzymes during fermentation (Chung et al., 2005). In addition, isovaleraldehyde was an important volatile compound in dry-cured ham products and fermented squid (Sabio et al., 1998; Huang L. D. et al., 2018). In addition, 1-octen-3-ol generated from lipid oxidation has been detected during soy sauce *koji* making (Feng et al., 2013) and is considered one of the most abundant compounds and important contributors to the sensory characteristics of *koji*.

**TABLE 1:** Free amino acid (FAA) profiles of samples taken from *koji* making fermentations at six different stages.*

FAA	Concentration (g/kg)					
	KJ0h	KJ6h	KJ12h	KJ24h	KJ36h	KJ48h
Phenylalanine	0.76 ± 0.03e	0.77 ± 0.01e	1.50 ± 0.02d	4.97 ± 0.13c	6.54 ± 0.48b	8.10 ± 0.32a
Leucine	0.80 ± 0.02e	0.79 ± 0.01e	1.63 ± 0.01d	4.92 ± 0.19c	6.00 ± 0.31b	6.66 ± 0.17a
Isoleucine	0.52 ± 0.01e	0.51 ± 0.01e	0.83 ± 0.01d	2.74 ± 0.07c	4.28 ± 0.24b	5.09 ± 0.12a
Tyrosine	0.45 ± 0.01e	0.47 ± 0.01e	0.83 ± 0.01d	2.22 ± 0.07c	3.40 ± 0.28b	4.52 ± 0.09a
Methionine	0.50 ± 0.02e	0.5 ± 0.03e	0.67 ± 0.03d	1.52 ± 0.02c	2.06 ± 0.11b	2.42 ± 0.06a
Valine	1.01 ± 0.07e	1.02 ± 0.01e	1.41 ± 0.04d	3.01 ± 0.05c	4.36 ± 0.19b	4.97 ± 0.13a
Proline	0.14 ± 0.01e	0.12 ± 0.01e	0.86 ± 0.04d	3.51 ± 0.06c	4.83 ± 0.30b	5.51 ± 0.19a
Alanine	0.41 ± 0.01e	0.29 ± 0.01e	1.02 ± 0.03d	3.84 ± 0.07c	4.5 ± 0.21b	5.74 ± 0.21a
Threonine	0.58 ± 0.04e	0.57 ± 0.01e	0.89 ± 0.01d	2.74 ± 0.07c	3.62 ± 0.26b	4.29 ± 0.18a
Serine	0.84 ± 0.01e	0.84 ± 0.01e	1.68 ± 0.03d	5.12 ± 0.05c	5.85 ± 0.46b	6.91 ± 0.29a
Glycine	0.14 ± 0.01e	0.15 ± 0.07e	0.34 ± 0.01d	1.72 ± 0.07c	2.60 ± 0.28b	2.94 ± 0.17a
Glutamic acid	2.19 ± 0.03d	2.02 ± 0.12de	1.85 ± 0.08e	4.53 ± 0.04c	5.42 ± 0.15b	5.85 ± 0.10a
Aspartic acid	1.44 ± 0.45d	1.71 ± 0.04d	1.75 ± 0.02d	6.74 ± 0.33c	9.21 ± 0.62b	10.42 ± 0.03a
Arginine	3.75 ± 0.01e	3.73 ± 0.25e	5.92 ± 0.02d	10 ± 0.15c	11.33 ± 0.63b	12.44 ± 0.18a
Lysine	1.09 ± 0.03d	1.04 ± 0.03d	3.37 ± 0.01c	8.62 ± 0.19b	8.45 ± 0.70b	10.10 ± 0.03a
Histidine	1.49 ± 0.21d	1.39 ± 0.03e	1.69 ± 0.03d	3.54 ± 0.02c	5.09 ± 0.19b	6.15 ± 0.06a

*Values represent means ± SD (n = 3). The concentration of each compound is reported as g/kg (dry weight). Different letters in the same row indicate statistically significant differences (p < 0.05).

### Dynamic Succession of Microbial Community Composition

**FIGURE 1:**
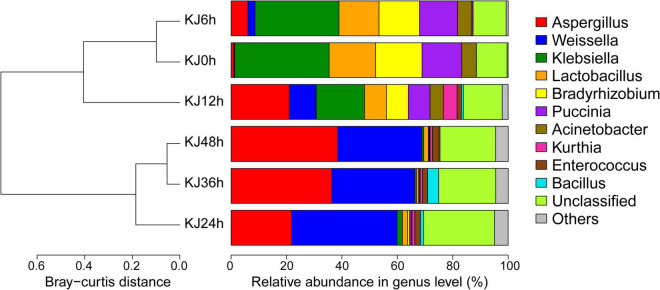
Taxonomic composition and community compositional clustering of *koji* metagenomes at the genus level among six fermentation stages spanning from 0 to 48 h. The 10-most abundant taxa are shown for all samples. “Others” comprise the less-abundant genus. Sequences that could not be assigned to known taxa were designated as “unclassified”. The cluster tree is based on the Bray-Curtis distance matrix of among-sample gene abundances.

To identify the succession of microbial communities present in *koji* making, metagenomic sequencing of six *koji* samples was conducted at different stages of production. Several large metagenomic data sets were produced from the *koji* samples, resulting in an average of 15.04 million 2 × 150 bp paired-end reads for each sample, and a total of 25.79 Gbp of sequence data after quality filtering (Supplementary Table 2). A large fraction of the sequence reads were assembled in contigs ≥500 bp (i.e., 358,780 contigs comprising 353,846,420 bp), yielding an N50 (defined as the contig length above which 50% of all assembled data are included) of 883, with a maximum contig length of 511,628 bp, and a mean contig size of 986 bp. Read-mapping of the quality-filtered reads to the assemblies was successful overall, with an average alignment rate of 67.03 ± 15.65% for the six samples (Supplementary Table 2). Thus, the assembled contigs possessed the majority of the sequenced genetic information for the *koji* microbial communities. Bacterial-associated sequences decreased in relative abundance from 79.17 to 59.70% during *koji* making while fungus-affiliated sequences increased from 20.83 to 40.30% (Supplementary Table 2). Thus, the ratio of fungal to bacterial sequences within communities during *Koji* making increased across stages, from 0.26 to 0.68. Archaeal-affiliated sequences were not observed in the *koji* metagenomes. UPGMA demonstrated that *koji* samples from the first three stages (0–12 h) belonged to one cluster, whereas later stage communities grouped into another cluster (Figure 1). *Firmicutes* (61.86 ± 7.52% relative abundances in the later three stages), *Ascomycota* (33.57 ± 9.48% in the later three stages), *Proteobacteria* (46.55 ± 13.23% in the first three stages), and *Basidiomycota* (12.06 ± 3.60% in the first three stages) were the dominant organisms throughout the various fermentation stages (Supplementary Figure 3). The relative abundances of the bacterial phylum *Proteobacteria* were dramatically higher in the early to middle stages (from 0 to 24 h; decreasing from 56.91 to 4.92%) as were those for the fungal phylum *Basidiomycota* (decreasing from 14.44 to 0.52%), while the relative abundances of the bacterial phylum *Firmicutes* and the fungal phylum *Ascomycota* increased across the entire fermentation period (0–48 h), with relative abundances increasing from 19.84 to 56.78% and from 1.02 to 40.06%, respectively (Supplementary Figure 3). The bacterial genera *Klebsiella*, *Lactobacillus*, and *Bradyrhizobium*, in addition to the fungal genus *Puccinia* dominated the beginning stage of fermentation (0 h), with overall abundances of 34.09, 16.78, 16.75, and 14.32%, respectively, but transitioned to minor populations after 24 h of fermentation (Figure 1). In contrast, the fungal genus *Aspergillus* and the bacterial genus *Weissella* exhibited increased abundances after 24 h of fermentation and then became dominant in the later *koji* making stages (24–48 h), with abundances reaching approximately 38.45 and 30.41% after 48 h of incubation. As expected, *Aspergillus* exhibited the highest overall abundance since it is used as a starter for soy sauce production (Yan et al., 2013). *Weissella* is a typical lactic acid bacterium (LAB) that is isolated and detected in a variety of fermented foods, and plays an important role in flavor generation (e.g., *via* production of lactic acid, isoamyl acetate, and terpinyl acetate) (Fessard and Remize, 2017; Xiang et al., 2020). *Weissella* has also previously been observed as one of the most dominant bacterial genera in soy sauce *koji* making (Yan et al., 2013). Additionally, the abundances of *Enterococcus* increased during fermentation, representing 2.42% of the microbial community at 48 h, but only 0.01% at 0 h. *Bacillus* was detected at low abundances at 24 and 36 h, accounting for 1.17 and 3.98% of community totals, respectively, but then decreased to 0.33% at 48 h (Figure 1). *Bacillus* spp. are the dominant microorganisms in many fermented soybean products (Devanthi and Gkatzionis, 2019; He and Chung, 2020) and their primary contributions to these systems is the production of various hydrolytic enzymes like proteases, α-amylases, and β-glucosidases that hydrolyze macromolecules (Sanjukta et al., 2015). *Bacillus* are thought to contribute to flavor generation during soy sauce aging *via* amylase and protease activities (Wei C. L. et al., 2013; Liang et al., 2019), but can also reduce the concentrations of allergens and improve the nutritional value of soy products (Shi et al., 2017). At the species level, *Klebsiella pneumoniae*, *Lactobacillus brevis*, *Bradyrhizobium* sp., and *Puccinia striiformis* were the predominant species in the initial stage of fermentation (0–12 h), while *A. oryzae*, Weissella *cibaria*, Weissella *confusa*, *Enterococcus italicus*, and *Bacillus subtilis* abundances gradually increased and became the dominant species in the middle to late stages (24–48 h) (Supplementary Table 3). Changes in microbiota composition also reflected the role of microorganisms during *koji* making. At the beginning of fermentation, most bacteria (e.g., *Klebsiella* and *Lactobacillus*) originate from raw materials (Yan et al., 2013). As fermentation proceeds, microbial compositions change, leading to substantial increases in *Aspergillus* and *Weissella* abundances. *Weissella* spp. are frequently detected in many spontaneously fermented foods and can adapt to diverse environments (Fessard and Remize, 2017). A high abundance of *Weissella* populations is likely to be associated with the generation of antimicrobial substances (e.g., bacteriocins) that may inhibit the growth of other bacteria (Fessard and Remize, 2017).

### Changes in Microbial Community Functional Potentials

**FIGURE 2:**
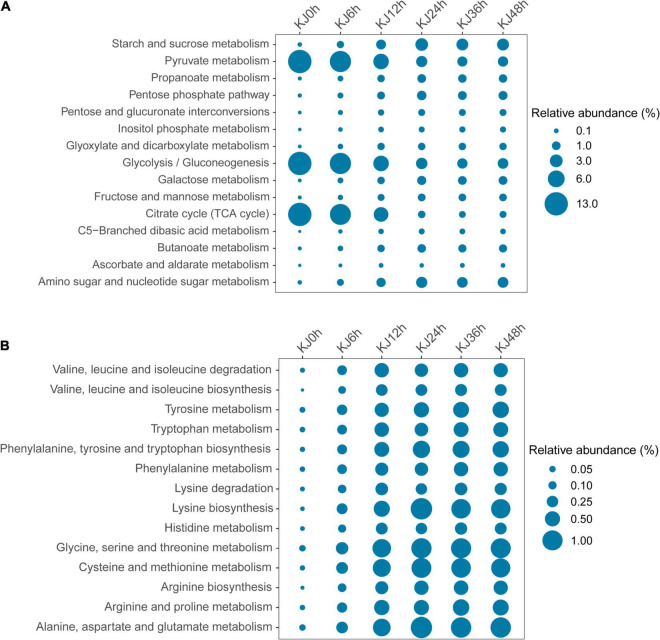
Functional profiling of carbohydrate metabolism **(A)** and amino acid metabolism **(B)** genes from whole-shotgun metagenome data that were classified at level 3 of the Kyoto Encyclopedia of Genes and Genomes (KEGG) annotation database.

**FIGURE 3:**
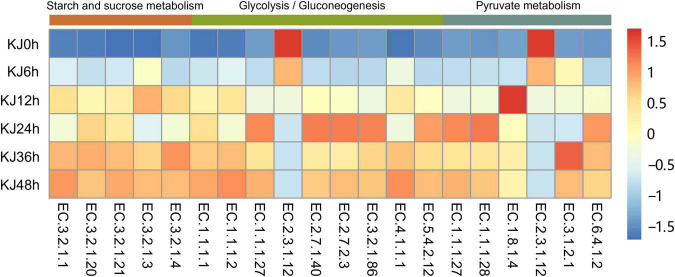
Changes in enzyme-encoding gene abundances among fermentation stages based on annotations against the Kyoto Encyclopedia of Genes and Genomes (KEGG) database within the carbohydrate metabolism category (e.g., starch and sucrose metabolism, glycolysis/gluconeogenesis, and pyruvate metabolism). Gene abundances were normalized by calculating Z scores of the tags per million (TPM) values for each fermentation stage. Heatmap values range from +1.5 to −1.5 and represent high abundance to low abundance levels.

Genes encoding metabolic functions dominated across the fermentation stages (increasing from 53.70 to 60.62%), followed by those involved in genetic information processing (decreasing from 23.34 to 17.21%), and environmental information processing (21.73–13.20%) based on KEGG annotations (Supplementary Figure 4). Gene abundances sharply changed at the 24 h fermentation point, then remained stable until the end of fermentation. The most represented metabolic gene sub-category was carbohydrate metabolism (relative abundance changes of 48.39–15.40%), followed by amino acid metabolism (1.05–9.68%), consistent with functional changes based on metagenomic analyses observed in soy sauce brines, sausage, and Chinese *paocai* (Sulaiman et al., 2014; Ferrocino et al., 2018; Liang et al., 2018; Kumar et al., 2019). Higher abundances of genes associated with carbohydrate and amino acid metabolism indicate that starches and proteins serve as critical flavor precursors. Within the carbohydrate metabolism category, genes involved in glycolysis/gluconeogenesis (ko00010, 9.52%), pyruvate metabolism (ko00620, average 9.49%), and the citrate cycle (TCA cycle) (ko00020, 9.17%) exhibited high abundances at the beginning of fermentation (e.g., in the 0, 6, and 12 h samples) that then decreased and remained stable as fermentation proceeded (Figure 2A and Supplementary Table 4). In the carbohydrate metabolism category, genes encoding dihydrolipoamide acetyltransferase (EC 2.3.1.12) within the glycolysis/gluconeogenesis pathway were detected in high abundance in the first two time points (0 and 6 h). Dihydrolipoamide acetyltransferase is a major component of the pyruvate dehydrogenase complex and is involved in pyruvic acid metabolism. The high observed abundances of genes encoding dihydrolipoamide acetyltransferases indicated a high activity of pyruvic acid biotransformation in the preliminary stage of *koji* making. The relative abundances then decreased throughout the fermentation process, although the abundances of other genes encoding L-lactate dehydrogenase (EC 1.1.1.27), pyruvate kinase (EC 2.7.1.40), and phosphoglycerate kinase (EC 2.7.2.3) increased from 0 to 24 h, but then slightly decreased (Figure 3). Decreases in the abundances of the gene encoding dihydrolipoamide acetyltransferase (EC 2.3.1.12), within the pyruvate metabolism category, were also observed. However, the relative abundances of genes encoding L-lactate dehydrogenase (EC 1.1.1.27), D-lactate dehydrogenase (EC 1.1.1.28), and acetyl-CoA hydrolase (EC 3.1.2.1) increased during the middle to late stages of fermentation (24–48 h) (Figure 3). Lactate dehydrogenase catalyzes the conversion of pyruvate to lactate with the oxidation of NADH to NAD^+^ (Schumann et al., 2002), while acetyl-CoA hydrolase catalyzes the production of acetate and corresponded to the increased concentrations of acetic acid that were measured during *koji* making (Supplementary Table 1). The above results suggest that the biotransformation of organic acids (e.g., lactate and acetate) in *koji* making derives from the metabolism of pyruvate and acetyl-CoA (from pyruvate), further suggesting that pyruvate is an important organic acid intermediate in the process. In addition, other genes related to carbohydrate metabolism also increased in abundance during fermentation. The abundances of genes involved in starch and sucrose metabolism (ko00500), in addition to amino sugar and nucleotide sugar metabolism (ko00520), increased from 0.08 to 2.50%, and from 0.07 to 1.89%, respectively (Figure 2A and Supplementary Table 4). In the middle to late stages of fermentation (24–48 h), starch and sucrose metabolic pathways (ko0500) were the most represented (average abundance of 2.63%), indicating that carbohydrates were likely used as an energy source during fermentation. Additional analysis of starch and sucrose metabolism revealed that the relative abundances of genes encoding alpha-amylase (EC 3.2.1.1) and glucoamylase (EC 3.2.1.3) steadily increased (Figure 3). Amylases and glucoamylases degrade starch molecules into simple sugars (e.g., glucose) that are useful energy sources for humans (Oguntoyinbo and Narbad, 2012; Tomasik and Horton, 2012). Starch and sucrose metabolism during fermentation are mainly related to *Aspergillus* taxa that are involved in the degradation of starch to glucose that is then utilized as a primary carbon source for microbial growth, while also contributing to the color and unique flavor profiles of fermentation products (Zhu et al., 2017). In contrast, genes involved in amino acid metabolism were present in low abundances prior to 12 h of fermentation, but then dramatically increased and remained stable at high abundances during the later stages (Figure 2B). The most abundant genes in the last three stages of fermentation were primarily associated with phenylalanine, tyrosine, and tryptophan biosynthesis (ko00400, average abundance of 0.65% in the last three stages); lysine biosynthesis (ko00300, 0.99%); glycine, serine, and threonine metabolism (ko00260, 1.00%); cysteine and methionine metabolism (ko00270, 0.93%); and alanine, aspartate and glutamate metabolism (ko00250, 1.06%) (Supplementary Table 4). These observations suggest that the *koji* microbiome exhibited a high potential for amino acid metabolism (biosynthesis), consistent with high FAA contents detected in the samples of this study (Table 1). Further, amino acids can be converted to various acids, alcohols (e.g., isoamyl alcohol), aldehydes (e.g., isovaleraldehyde, 2-methylbutyraldehyde, benzaldehyde, and phenylacetaldehyde), and esters (e.g., methyl acetate and ethyl acetate) (Supplementary Table 1), all of which may contribute to the flavor development of *koji*. In particular, increases in the relative abundances of genes (0.03–0.43%) were observed related to valine, leucine, and isoleucine degradation (ko00280) during *koji* making. This metabolic pathway involves the three branched-chain amino acids (BCAA; isoleucine, leucine, and valine) that have been reported as precursors to volatile compounds like acids, alcohols, and esters (Marilley and Casey, 2004; Smit et al., 2005).

### Correlations Between Microbiota and Metabolites

**FIGURE 4:**
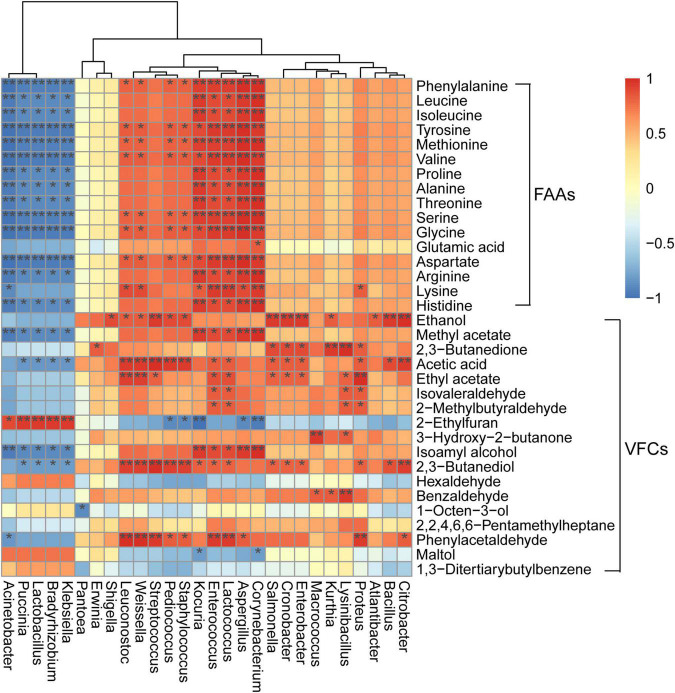
Heatmap of correlations among microbial genera and metabolites. Correlation strength (based on Spearman’s *r*-value) and correlation significance values are shown as shaded colors (red, positive correlation; blue, negative correlation). Heatmap values range from +1.0 to −1.0. Values above/below zero represent positive/negative correlations, respectively, between genera and parameters. *p < 0.05, **p < 0.01.

Correlations between microbial populations and flavor profiles have been reported for *moromi* fermentation, wherein *Lactococcus* and *Weissella* abundances were positively correlated with oxalic acid concentrations (Liu et al., 2021). However, these relationships have not been evaluated for the *koji* making process. Here, the abundances of *Aspergillus*, *Kocuria*, *Corynebacterium*, *Enterococcus*, and *Lactococcus* were positively correlated with the concentrations of all amino acids (except for glutamic acid) (all *p* < 0.05) (Figure 4), indicating potential roles of these genera in the production and transformation of amino acids in *koji* making. In addition, the abundances of *Acinetobacter*, *Puccinia*, *Bradyrhizobium*, *Klebsiella*, and *Lactobacillus* were negatively associated with the contents of the metabolites mentioned above and positively with 2-ethylfuran (all *p* < 0.05). *A. oryzae* is a common starter that is inoculated into soy sauce *koji* and is capable of secreting proteases to hydrolyze proteins and promote the production of peptides and amino acids (Vishwanatha et al., 2009; Gao et al., 2019). Further, *Enterococcus* is one of the main producers of methyl and ethyl esters due to its observed esterase and lipase activities in food fermentation (Jin et al., 2019). Moreover, *Enterococcus* abundances have also been positively correlated with the concentrations of 16 amino acids in the fermented soybean food sufu (Huang X. N. et al., 2018). This study also demonstrated the positive correlation of *Enterococcus* abundances to concentrations of all 16 amino acids that were analyzed, with 15 exhibiting significantly positive correlations to abundances (all *p* < 0.05).

The abundances of the low-abundance genus *Corynebacterium* (<1% relative abundance at each stage) were also positively correlated to the concentrations of all 16 amino acids and two VFCs (methyl acetate and isoamyl alcohol) (all *p* < 0.05), suggesting a possible role in flavor generation. *Weissella* was positively correlated to the concentrations of eight amino acids (phenylalanine, tyrosine, methionine, valine, serine, glycine, aspartate, and lysine), phenylacetaldehyde, acetic acid, 2,3-butanediol, ethyl acetate, and ethanol (all *p* < 0.05). Additionally, the abundances of other genera (such as *Streptococcus*, *Bacillus*, *Cronobacter*, *Pediococcus*, and *Kurthia*) also exhibited positive relationships with ethanol concentrations (all *p* < 0.05) (Figure 4), indicating that these genera, may contribute to the production of ethanol. It is worth noting that these correlational results between microbial populations and metabolites in *koji* were from fermentation that used whole soybeans. However, different soybean materials may influence microbial communities and the associations between microorganism abundances and metabolites. Therefore, further studies are needed to evaluate these interactions in *koji*.

## Conclusion

In this study, changes in microbial community compositions, genetic functions, and metabolites were investigated during *koji* making by coupling shotgun metagenomics and metabolomics. To the best of our knowledge, this is the first study to comprehensively evaluate the functional potentials of microbial communities and correlations between microbiota and metabolites during *koji* making. *Aspergillus* and *Weissella* were identified as the most abundant microbial taxa during *koji* making. In addition, functional profiling analysis indicated that metabolic functions of the *koji* microbiome exhibited drastic shifts after 24 h of fermentation, wherein the abundances of genes related to pyruvate metabolism, glycolysis/gluconeogenesis, and the TCA cycle greatly decreased, while the abundances increased for other functional genes associated with carbohydrate and amino acid metabolism. Among the latter category, genes that were enriched in later stages of fermentation included those involved in starch and sucrose metabolism; amino sugar and nucleotide sugar metabolism; alanine, aspartate, and glutamate metabolism; glycine, serine, and threonine metabolism; lysine biosynthesis; and cysteine and methionine metabolism. Correlational analyses indicated that the abundances of *Aspergillus*, *Kocuria, Enterococcus*, *Lactococcus*, and *Corynebacterium* were all positively correlated with the concentrations of all amino acids (except for glutamic acid), while *Weissella* abundances were positively associated with the concentrations of eight amino acids, phenylacetaldehyde, acetic acid, 2,3-butanediol, ethyl acetate, and ethanol. Overall, this study provides novel insights into the roles of microbial communities in the generation of metabolites during *koji* making. A better understanding of the microbial taxonomic and functionality within *koji* can help optimize product quality and further improve the flavor of soy sauce products. Lastly, *Aspergillus* and *Weissella* populations were frequently detected in similar high abundance during *koji* making, but little is known about their potential inter-species interactions. Future studies should be conducted to investigate potential coexistence relationships between these two species during soy sauce manufacturing.

## Data Availability Statement

The datasets presented in this study can be found in online repositories. The names of the repository/repositories and accession number(s) can be found in the article/Supplementary Material.

## Author Contributions

GT and MH conceived and designed the experiments in addition to writing the manuscript. GT and YW conducted the experiments and data analyses. XiL, XuL, and ZP performed most of the experiments, while ML, LL, and ZZ supervised the execution of the experiments. All authors read and approved the final manuscript.

## Conflict of Interest

The authors declare that the research was conducted in the absence of any commercial or financial relationships that could be construed as a potential conflict of interest.

## Publisher’s Note

All claims expressed in this article are solely those of the authors and do not necessarily represent those of their affiliated organizations, or those of the publisher, the editors and the reviewers. Any product that may be evaluated in this article, or claim that may be made by its manufacturer, is not guaranteed or endorsed by the publisher.

## Supplementary Material

Supplementary Figure 1 *Koji* making overview and sampling times.

Supplementary Figure 2 pH changes during *koji* making.

Supplementary Figure 3 Taxonomic composition of *koji* metagenomes at the phylum level showing changes in microbial communities across six fermentation stages (0–48 h). Sequences that could not be classified to known taxa were designated as “unclassified”.

Supplementary Figure 4 Functional profiles produced from whole shotgun metagenome-derived ORFs annotated at level 2 of the Kyoto Encyclopedia of Genes and Genomes (KEGG) database.
