# Supplementary material for: Microbial Community and Metabolite Dynamics During Soy Sauce Koji Making
Source: Front Microbiol. 2022 Feb 25;13:841529. doi: 10.3389/fmicb.2022.841529 (PMC8914375; doi:10.3389/fmicb.2022.841529)
Supplement: Supplementary file 1 [file Data_Sheet_1.docx]

**Supplementary Tables for**

Microbial Community and Metabolite Dynamics During Soy Sauce *Koji* Making

**Guiliang Tan^a^, Min Hu^b*^, Xiangli Li^c^, Xueyan Li^a^, Ziqiang Pan^a^, Mei Li^a^, Lin Li^a^, Yi Wang^a,*^, Ziyi Zheng^a^**

^a^ *School of Material Science and Food Engineering, University of Electronic Science and Technology of China, Zhongshan Institute, Zhongshan 528402, China;*

^b^ *School of Environmental and Safety Engineering, Changzhou University, Changzhou 213164, China;*

^c^ *School of Health Industry, Zhongshan Torch Vocational and Technical College, Zhongshan 528436, China.*

∗ *Correspondence to: Min Hu, School of Environmental and Safety Engineering, Changzhou University, Changzhou 213164, China. E-mail: humin@soil.gd.cn*. *Yi Wang,* *School of Material Science and Food Engineering, University of Electronic Science and Technology of China, Zhongshan Institute, Zhongshan 528402, China. E-mail: wangyi@zsc.edu.cn.*

**Table S1.** VFC profiles of samples taken from *koji* making fermentations at six different stages.

| **VFCs** | **Concentration (μg/kg)** | | | | | |
| --- | --- | --- | --- | --- | --- | --- |
|  | **KJ0h** | **KJ6h** | **KJ12h** | **KJ24h** | **KJ36h** | **KJ48h** |
| **Esters (12)** |  |  |  |  |  |  |
| Methyl acetate | 79.95 ± 6.55e | 67.12 ± 2.13f | 138.65 ± 4.25d | 179.02 ± 6.35c | 186.86 ± 6.46b | 422.03 ± 36.28a |
| Ethyl acetate | / | / | 117.17 ± 2.31b | 135.96 ± 4.27a | 46.88 ± 5.57c | 130.91 ± 8.42a |
| Methyl butyrate | 6.83 ± 0.15b | 7.08 ± 1.05b | / | / | / | 11.12 ± 2.23a |
| Methyl isovalerate | / | / | / | / | / | 53.43 ± 0.90 |
| Ethyl isovalerate | / | / | / | 23.18 ± 2.99b |  | 49.89 ± 7.22a |
| Isoamyl acetate | / | / | / | / | / | 132.13 ± 3.56 |
| Methyl hexanoate | / | / | / | / | 9.84 ± 1.46b | 27.80 ± 5.75a |
| Methyl benzoate | / | / | / | 4.79 ± 0.33b | 6.54 ± 0.91b | 11.09 ±3.44a |
| Methyl octanoate | / | / | / | 7.97 ± 0.58b | 9.2 ± 1.13b | 21.09 ± 5.77a |
| Methyl hexadecanoate | 4.27 ± 0.99d | / | / | 12.50 ± 2.04c | 30.82 ± 1.56b | 90.45 ± 11.21a |
| Ethyl palmitate | / | / | / | / | 3.43 ± 0.05b | 9.22 ± 3.66a |
| Methyl linoleate | / | / | / | / | 20.63 ± 2.90b | 57.50 ± 7.45a |
| **Alcohols (10)** |  |  |  |  |  |  |
| Ethanol | 52.00 ± 2.82d | 52.41 ± 1.22d | 118.2 ± 1.80b | 131.96 ± 14.92a | 120.69 ± 8.15b | 99.38 ± 9.46c |
| Isoamyl alcohol | 26.67 ± 1.69e | 25.19 ± 1.51e | 65.96 ± 2.10d | 101.12 ± 2.91c | 123.89 ± 11.39b | 276.65 ± 16.12a |
| Pentyl alcohol | 34.14 ± 1.78a | 25.24 ± 0.95b | / | / | / | / |
| 2,3-Butanediol | / | / | 26.51 ± 0.50d | 250.63 ± 8.22a | 104.05 ± 2.86b | 36.63 ± 4.69c |
| (R,R)-2,3-Butanediol | / | / | 47.36 ± 6.84b | / | / | 101.58 ± 14.03a |
| Hexyl alcohol | 101.02 ± 8.85a | 84.27 ± 2.90b | 44.05 ± 0.72c | / | / | / |
| 1-Octen-3-ol | 320.91 ± 14.99c | 298.24 ± 12.43d | 354.49 ± 3.68b | 177.08 ± 7.72e | 154.39 ± 10.88f | 385.46 ± 0.38a |
| 3-Octanol | 6.23 ± 1.91a | 6.21 ± 1.27a | / | / | / | / |
| Maltol | 83.17 ± 1.78c | 93.60 ± 2.81b | 133.59 ± 4.21a | 62.68 ± 4.47d | / | / |
| Phenethyl alcohol | / | / | / | / | 27.01 ± 5.48b | 36.77 ± 1.93a |
| **Aldehydes (10)** |  |  |  |  |  |  |
| Acetaldehyde | / | / | 33.35 ± 5.93 | / | / | / |
| Isobutyraldehyde | / | 26.79 ± 4.25d | 63.71 ± 3.61b | / | 38.23 ± 5.52c | 88.36 ± 14.10a |
| Isovaleraldehyde | 19.02 ± 3.12f | 60.83 ± 2.62e | 316.24 ± 2.61b | 248.89 ±32.04c | 189.21 ± 7.63d | 939.75 ± 27.4a |
| 2-Methylbutyraldehyde | / | 40.75 ± 0.62d | 100.13 ± 0.23b | 84.75 ± 1.94c | 82.89 ± 12.1c | 197.14 ± 8.58a |
| Hexaldehyde | 206.92 ± 8.48c | 238.35 ± 12.85b | 306.27 ± 4.28a | 153.71 ± 7.98d | 133.08 ± 3.59e | 159.69 ± 10.83d |
| 3-Methylthiopropoanaldehyde | / | / | 17.91 ± 0.10 c | 27.99 ± 0.22b | 29.22 ± 0.47b | 51.02 ± 0.96a |
| Benzaldehyde | 24.36 ± 2.66e | 28.23 ± 1.97e | 134.26 ± 2.47a | 45.91 ± 4.89c | 39.94 ± 4.29d | 100.26 ± 8.30b |
| Phenylacetaldehyde | / | 5.61 ± 1.18e | 43.82 ± 0.81d | 147.58 ± 5.36a | 70.80 ± 4.10c | 113.80 ± 5.52b |
| Nonyl aldehyde | / | / | 11.13 ± 0.09a | 6.36 ± 0.12c | 8.54 ± 0.22b | / |
| 2-Phenylcrotonaldehyde | / | / | 10.55 ± 0.17 c | 12.33 ± 0.21bc | 15.98 ± 0.55b | 28.04 ± 0.31a |
| **Ketones (6)** |  |  |  |  |  |  |
| Acetone | 81.05 ± 3.56 | / | / | / | / | / |
| 2,3-Butanedione | / | 12.72 ± 1.72d | 236.84 ± 4.74a | 78.91 ± 2.76b | 46.80 ± 4.10c | 47.08 ± 6.31c |
| 2-Butanone | 21.51 ± 0.12b | 20.95 ± 0.23b | / | / | / | 42.26 ± 0.19a |
| 2-Pentanone | 13.12 ± 0.09c | 26.52 ± 0.47a | 15.82 ± 0.32b | 16.15 ± 0.58b | / | / |
| 3-Hydroxy-2-butanone | / | 35.78 ± 2.42e | 282.48 ± 7.20a | 80.62 ± 8.05d | 90.52 ± 6.00c | 108.72 ± 6.56b |
| 2-Heptanone | / | 31.09 ± 2.41 | / | / | / | / |
| **Acids (4)** |  |  |  |  |  |  |
| Acetic acid | 31.67 ± 2.63d | 36.33 ± 1.42d | 152.27 ± 8.26c | 221.26 ± 6.18a | 169.81 ± 30.47b | 155.62 ± 10.17c |
| Isovaleric acid | / | / | / | / | 53.82 ± 0.55b | 98.02 ± 1.35 a |
| Heptanoic acid | / | / | / | / | 52.09 ± 1.02 | / |
| 2-Methyl butyric acid |  |  | / | / | 56.12 ± 2.33b | 81.06 ± 0.75a |
| **Others (24)** |  |  |  |  |  |  |
| Pentane | / | / | / | 56.33 ± 1.46 | / | / |
| Ethyl vinyl ether | / | / | / | 47.41 ± 0.99 | / | / |
| 2-Ethylfuran | 109.36 ± 12.73a | 114.3 ± 4.35a | 104.54 ± 3.60a | 65.43 ± 8.18b | 33.20 ± 1.98c | 64.98 ± 0.72b |
| Toluene | 13.97 ± 0.23d | 13.8 ± 0.49d | 24.45 ± 1.68 bc | 26.14 ± 0.55b | 20.50 ± 0.34c | 43.64 ± 1.01a |
| 3-Methyl-Heptane | / | / | 29.62 ± 0.52b | 19.53 ± 0.23c | 19.76 ± 0.56c | 46.09 ± 0.83a |
| 2,4-Dimethyl-heptane | 8.99 ± 0.13bc | 8.14 ± 0.36c | 8.18 ± 0.05c | 9.89 ± 0.20b | 6.22 ± 0.03d | 20.88 ± 2.55a |
| Ethylbenzene | 29.38 ± 0.15b | 25.05 ± 0.32c | 27.38 ± 0.19bc | 28.42 ± 1.07bc | 19.12 ± 1.66d | 39.91 ± 0.67a |
| 4-Methyloctane | 10.46 ± 0.23de | 9.42 ± 0.09 e | 13.48 ± 0.83d | 25.16 ± 1.54b | 19.35 ± 0.61c | 48.11 ± 0.99a |
| 1,3-Dimethyl-Benzene | / | 25.69b | 37.44a | / | / | / |
| 1,4-Dimethyl-Benzene | 31.1 ± 0.91c | / | / | 39.31 ± 0.55b | 37.30 ± 0.98b | 77.31 ± 2.43a |
| 2-Methyl-1-pentene | / | / | / | 37.49 ± 2.06 | / | / |
| Phenylethylene | 14.71 ± 0.06b | 14.14 ± 0.25b | / | 38.52 ± 0.37a |  | / |
| n-Nonane | 12.92 ± 0.22c | 16.59 ± 0.71ab | 19.68 ± 0.52a | 15.8 ± 0.13bc | 14.88 ± 0.64bc | / |
| 4-Ethyloctane |  |  |  | / | 6.87 ± 0.11b | 14.97 ± 0.23a |
| 3-Methylnonane | 12.93 ± 0.13c | 13.71 ± 0.36c | 16.62 ± 0.49b | 16.84 ±0.09 b | 11.81 ± 0.37c | 23.12 ± 1.62a |
| 2,2,4,6,6-Pentamethylheptane | 149.33 ± 4.20e | 243.97 ± 11.12c | 307.9 ± 9.26b | 304.89 ± 4.87b | 231.07 ±147.83d | 470.53 ± 20.13a |
| Decane | 45.33 ± 1.01 d | 46.75 ± 0.64d | 56.59 ± 0.42b | 51.78 ± 2.97c | 40.94 ± 3.12e | 83.42 ± 1.09a |
| 4-Bromoheptane | 20.06 ± 0.33b | / | 34.93 ± 0.29a | / | / | / |
| 2,2,4,4,6,8,8-heptamethyl-Nonane | / | / | / | / | 45.31 ± 2.78b | 106.29 ± 3.42a |
| 2,2,4,4-Tetramethyloctane | / | 27.21± 0.47b | / | 47.47 ± 0.98a | / | / |
| 5-Methylundecane | 9.69 ± 0.39b | / | / | / | / | 23.55 ± 0.67a |
| 3-Methylundecane | / | 6.66 ± 0.09d | 9.52 ± 0.12b | 7.40 ± 0.67c | 6.06 ± 0.44d | 12.33 ± 0.25a |
| Dodecane | 9.29 ± 0.17c | 7.00 ± 0.10d | 11.57 ± 0.09b | 11.40 ± 0.38b | 5.70 ± 0.05e | 13.48 ± 0.19a |
| 1,3-Ditertiarybutylbenzene | 123.27 ± 10.60b | 85.72 ± 2.09c | 150.95 ± 3.41a | 79.91 ± 3.60d | 72.09 ± 9.84e | 121.16 ± 1.53b |

^*^ Values represent means ± SD (n=3). The concentration of each compound is shown in μg/kg (dry weight). Different letters in the same row indicate statistically significant differences (*p* < 0.05).

**Table S2**. Sequencing and assembly information in addition to taxonomic composition of *koji* samples^*^.

| Metagenomic information | KJ0h | KJ6h | KJ12h | KJ24h | KJ36h | KJ48h | Mixed assembly |
| --- | --- | --- | --- | --- | --- | --- | --- |
| Total raw sequences | 12,850,782 | 12,159,719 | 14,909,774 | 14,201,912 | 12,102,607 | 24,039,388 |  |
| Total clean sequences | 12,002,331 | 11,478,977 | 14,255,871 | 13,462,718 | 11,565,206 | 23,199,995 |  |
| Total sequence length | 3.6 Gbp | 3.44 Gbp | 4.28 Gbp | 4.04 Gbp | 3.47 Gbp | 6.96 Gbp |  |
| % GC | 46 | 46 | 46 | 45 | 46 | 46 |  |
| Q20 (%) | 100 | 100 | 100 | 100 | 100 | 100 |  |
| Q30 (%) | 99.4 | 99.65 | 99.49 | 99.55 | 99.64 | 99.51 |  |
| Alignment rate (%) | 49.47 | 52.78 | 57.89 | 73.79 | 84.18 | 84.05 |  |
| No. of ORFs | 15,571 | 32,440 | 67,734 | 91,847 | 88,937 | 94,225 |  |
| Bacteria (%) | 79.17 | 74.87 | 67.61 | 76.53 | 61.85 | 59.70 |  |
| Fungi (%) | 20.83 | 25.13 | 32.39 | 23.47 | 38.15 | 40.30 |  |
| Archaea (%) | 0.00 | 0.00 | 0.00 | 0.00 | 0.00 | 0.00 |  |
| Fungi/Bacteria | 0.26 | 0.34 | 0.48 | 0.31 | 0.62 | 0.68 |  |
| No. of contigs |  |  |  |  |  |  | 358,780 |
| Max. contig length |  |  |  |  |  |  | 511,628 bp |
| Min. contig length |  |  |  |  |  |  | 500 bp |
| N50 |  |  |  |  |  |  | 883 |

^*^ Metagenomic data were taxonomically profiled using MEGAN based on BLASTX analysis against the NCBI-nr database.

**Table S3**. Microbial species relative abundances (%) during *koji* fermentation.

| **Genus** | **Species** | **KJ0h** | **KJ6h** | **KJ12h** | **KJ24h** | **KJ36h** | **KJ48h** |
| --- | --- | --- | --- | --- | --- | --- | --- |
| ***Aspergillus*** | *Aspergillus flavus* | 0.04 | 0.23 | 0.79 | 0.83 | 1.41 | 1.48 |
|  | *Aspergillus oryzae* | 0.04 | 0.27 | 0.90 | 0.92 | 1.62 | 1.69 |
|  | unclassified *Aspergillus* | 0.90 | 5.57 | 19.29 | 20.09 | 33.31 | 35.27 |
| ***Weissella*** | *Weissella cibaria* | 0.08 | 0.34 | 1.51 | 8.12 | 7.26 | 7.60 |
|  | *Weissella confusa* | 0.07 | 1.07 | 3.67 | 12.43 | 9.59 | 8.88 |
|  | *Weissella hellenica* | 0.00 | 0.00 | 0.01 | 0.04 | 0.03 | 0.03 |
|  | *Weissella jogaejeotgali* | 0.00 | 0.00 | 0.02 | 0.05 | 0.06 | 0.05 |
|  | *Weissella paramesenteroides* | 0.02 | 0.01 | 0.20 | 0.77 | 0.56 | 0.61 |
|  | unclassified *Weissella* | 0.14 | 1.14 | 4.34 | 16.65 | 12.42 | 13.24 |
| ***Klebsiella*** | *Klebsiella pneumoniae* | 34.09 | 30.29 | 17.35 | 1.83 | 0.47 | 0.66 |
|  | unclassified *Klebsiella* | 0.00 | 0.02 | 0.04 | 0.09 | 0.14 | 0.02 |
| ***Lactobacillus*** | *Lactobacillus brevis* | 16.77 | 14.41 | 7.71 | 0.96 | 0.11 | 0.47 |
|  | *Lactobacillus dextrinicus* | 0.00 | 0.00 | 0.00 | 0.00 | 0.05 | 0.01 |
|  | *Lactobacillus fermentum* | 0.00 | 0.00 | 0.01 | 0.02 | 0.02 | 0.02 |
|  | *Lactobacillus manihotivorans* | 0.00 | 0.00 | 0.02 | 0.07 | 0.02 | 0.28 |
|  | *Lactobacillus plantarum* | 0.00 | 0.00 | 0.01 | 0.03 | 0.02 | 0.12 |
|  | *Lactobacillus spicheri* | 0.00 | 0.00 | 0.00 | 0.00 | 0.00 | 0.01 |
|  | unclassified *Lactobacillus* | 0.01 | 0.03 | 0.16 | 0.82 | 0.61 | 0.78 |
| ***Bradyrhizobium*** | *Bradyrhizobium* sp. MOS002 | 16.75 | 14.67 | 7.94 | 0.68 | 0.11 | 0.25 |
| ***Puccinia*** | *Puccinia striiformis* | 11.43 | 11.01 | 6.29 | 0.43 | 0.08 | 0.11 |
|  | unclassified *Puccinia* | 2.89 | 2.69 | 1.55 | 0.11 | 0.02 | 0.03 |
| ***Enterococcus*** | *Enterococcus faecium* | 0.00 | 0.00 | 0.02 | 0.04 | 0.03 | 0.04 |
|  | *Enterococcus italicus* | 0.01 | 0.01 | 1.08 | 1.28 | 1.19 | 1.55 |
|  | *Enterococcus pseudoavium* | 0.00 | 0.00 | 0.01 | 0.01 | 0.01 | 0.02 |
|  | unclassified *Enterococcus* | 0.00 | 0.01 | 0.43 | 0.69 | 0.59 | 0.81 |
| ***Bacillus*** | *Bacillus subtilis* | 0.00 | 0.00 | 0.02 | 0.17 | 0.35 | 0.02 |
|  | *Bacillus velezensis* | 0.00 | 0.00 | 0.02 | 0.02 | 0.07 | 0.00 |
|  | unclassified *Bacillus* | 0.02 | 0.09 | 0.68 | 0.97 | 3.56 | 0.30 |
| ***Kurthia*** | *Kurthia huakuii* | 0.00 | 0.08 | 1.23 | 0.22 | 0.15 | 0.19 |
|  | *Kurthia massiliensis* | 0.00 | 0.01 | 0.14 | 0.03 | 0.02 | 0.02 |
|  | *Kurthia senegalensis* | 0.00 | 0.01 | 0.08 | 0.02 | 0.01 | 0.01 |
|  | *Kurthia* sp. 11kri321 | 0.00 | 0.02 | 0.08 | 0.02 | 0.03 | 0.02 |
|  | *Kurthia* sp. Dielmo | 0.00 | 0.03 | 0.46 | 0.09 | 0.06 | 0.07 |
|  | *Kurthia zopfii* | 0.00 | 0.01 | 0.05 | 0.02 | 0.02 | 0.01 |
|  | unclassified *Kurthia* | 0.01 | 0.34 | 2.89 | 0.64 | 0.57 | 0.52 |
| ***Acinetobacter*** | *Acinetobacter baumannii* | 5.24 | 4.87 | 2.87 | 0.20 | 0.04 | 0.05 |
|  | *Acinetobacter defluvii* | 0.00 | 0.00 | 0.11 | 0.01 | 0.02 | 0.01 |
|  | unclassified *Acinetobacter* | 0.02 | 0.10 | 1.86 | 0.15 | 0.26 | 0.14 |

**Table S4**. Relative abundances (%) of genes throughout *koji* making stages at the KEGG level 3 functional within categories of carbohydrate metabolism and amino acid metabolism.

|  | **Level 3** | **Level 2** | **KJ0h** | **KJ6h** | **KJ12h** | **KJ24h** | **KJ36h** | **KJ48h** |
| --- | --- | --- | --- | --- | --- | --- | --- | --- |
| ko00520 | Amino sugar and nucleotide sugar metabolism | Carbohydrate metabolism | 0.07 | 0.49 | 1.25 | 2.10 | 1.84 | 1.89 |
| ko00053 | Ascorbate and aldarate metabolism | Carbohydrate metabolism | 0.01 | 0.03 | 0.10 | 0.07 | 0.11 | 0.09 |
| ko00650 | Butanoate metabolism | Carbohydrate metabolism | 0.04 | 0.21 | 0.62 | 0.95 | 0.84 | 0.84 |
| ko00660 | C5-Branched dibasic acid metabolism | Carbohydrate metabolism | 0.01 | 0.06 | 0.19 | 0.28 | 0.24 | 0.23 |
| ko00020 | Citrate cycle (TCA cycle) | Carbohydrate metabolism | 13.06 | 10.19 | 4.27 | 0.62 | 0.45 | 0.49 |
| ko00051 | Fructose and mannose metabolism | Carbohydrate metabolism | 0.03 | 0.18 | 0.50 | 0.71 | 0.68 | 0.69 |
| ko00052 | Galactose metabolism | Carbohydrate metabolism | 0.03 | 0.26 | 0.63 | 1.17 | 1.03 | 1.05 |
| ko00010 | Glycolysis / Gluconeogenesis | Carbohydrate metabolism | 13.10 | 10.49 | 4.97 | 2.22 | 1.65 | 1.83 |
| ko00630 | Glyoxylate and dicarboxylate metabolism | Carbohydrate metabolism | 0.03 | 0.20 | 0.60 | 0.62 | 0.62 | 0.60 |
| ko00562 | Inositol phosphate metabolism | Carbohydrate metabolism | 0.02 | 0.08 | 0.23 | 0.28 | 0.32 | 0.30 |
| ko00040 | Pentose and glucuronate interconversions | Carbohydrate metabolism | 0.02 | 0.14 | 0.34 | 0.49 | 0.47 | 0.49 |
| ko00030 | Pentose phosphate pathway | Carbohydrate metabolism | 0.05 | 0.28 | 0.73 | 1.33 | 1.06 | 1.12 |
| ko00640 | Propanoate metabolism | Carbohydrate metabolism | 0.04 | 0.25 | 0.63 | 1.03 | 0.94 | 0.92 |
| ko00620 | Pyruvate metabolism | Carbohydrate metabolism | 13.09 | 10.44 | 4.95 | 2.10 | 1.57 | 1.67 |
| ko00500 | Starch and sucrose metabolism | Carbohydrate metabolism | 0.08 | 0.58 | 1.47 | 2.93 | 2.45 | 2.50 |
| ko00250 | Alanine, aspartate and glutamate metabolism | Amino acid metabolism | 0.05 | 0.26 | 0.73 | 1.13 | 1.02 | 1.03 |
| ko00330 | Arginine and proline metabolism | Amino acid metabolism | 0.03 | 0.18 | 0.51 | 0.50 | 0.62 | 0.57 |
| ko00220 | Arginine biosynthesis | Amino acid metabolism | 0.02 | 0.12 | 0.34 | 0.44 | 0.45 | 0.43 |
| ko00270 | Cysteine and methionine metabolism | Amino acid metabolism | 0.03 | 0.27 | 0.75 | 0.97 | 0.94 | 0.88 |
| ko00260 | Glycine, serine and threonine metabolism | Amino acid metabolism | 0.05 | 0.31 | 0.82 | 1.01 | 1.00 | 0.99 |
| ko00340 | Histidine metabolism | Amino acid metabolism | 0.02 | 0.10 | 0.27 | 0.24 | 0.30 | 0.27 |
| ko00300 | Lysine biosynthesis | Amino acid metabolism | 0.02 | 0.21 | 0.58 | 1.15 | 0.92 | 0.91 |
| ko00310 | Lysine degradation | Amino acid metabolism | 0.02 | 0.11 | 0.32 | 0.24 | 0.31 | 0.29 |
| ko00360 | Phenylalanine metabolism | Amino acid metabolism | 0.03 | 0.16 | 0.35 | 0.37 | 0.44 | 0.44 |
| ko00400 | Phenylalanine, tyrosine and tryptophan biosynthesis | Amino acid metabolism | 0.03 | 0.17 | 0.48 | 0.69 | 0.66 | 0.61 |
| ko00380 | Tryptophan metabolism | Amino acid metabolism | 0.03 | 0.16 | 0.45 | 0.38 | 0.48 | 0.49 |
| ko00350 | Tyrosine metabolism | Amino acid metabolism | 0.04 | 0.19 | 0.43 | 0.51 | 0.56 | 0.61 |
| ko00290 | Valine, leucine and isoleucine biosynthesis | Amino acid metabolism | 0.02 | 0.08 | 0.26 | 0.26 | 0.28 | 0.27 |
| ko00280 | Valine, leucine and isoleucine degradation | Amino acid metabolism | 0.03 | 0.16 | 0.44 | 0.37 | 0.44 | 0.43 |
