# Supplementary figures and images for: Microbial Community and Metabolite Dynamics During Soy Sauce Koji Making
Source: Front Microbiol. 2022 Feb 25;13:841529. doi: 10.3389/fmicb.2022.841529 (PMC8914375; doi:10.3389/fmicb.2022.841529)

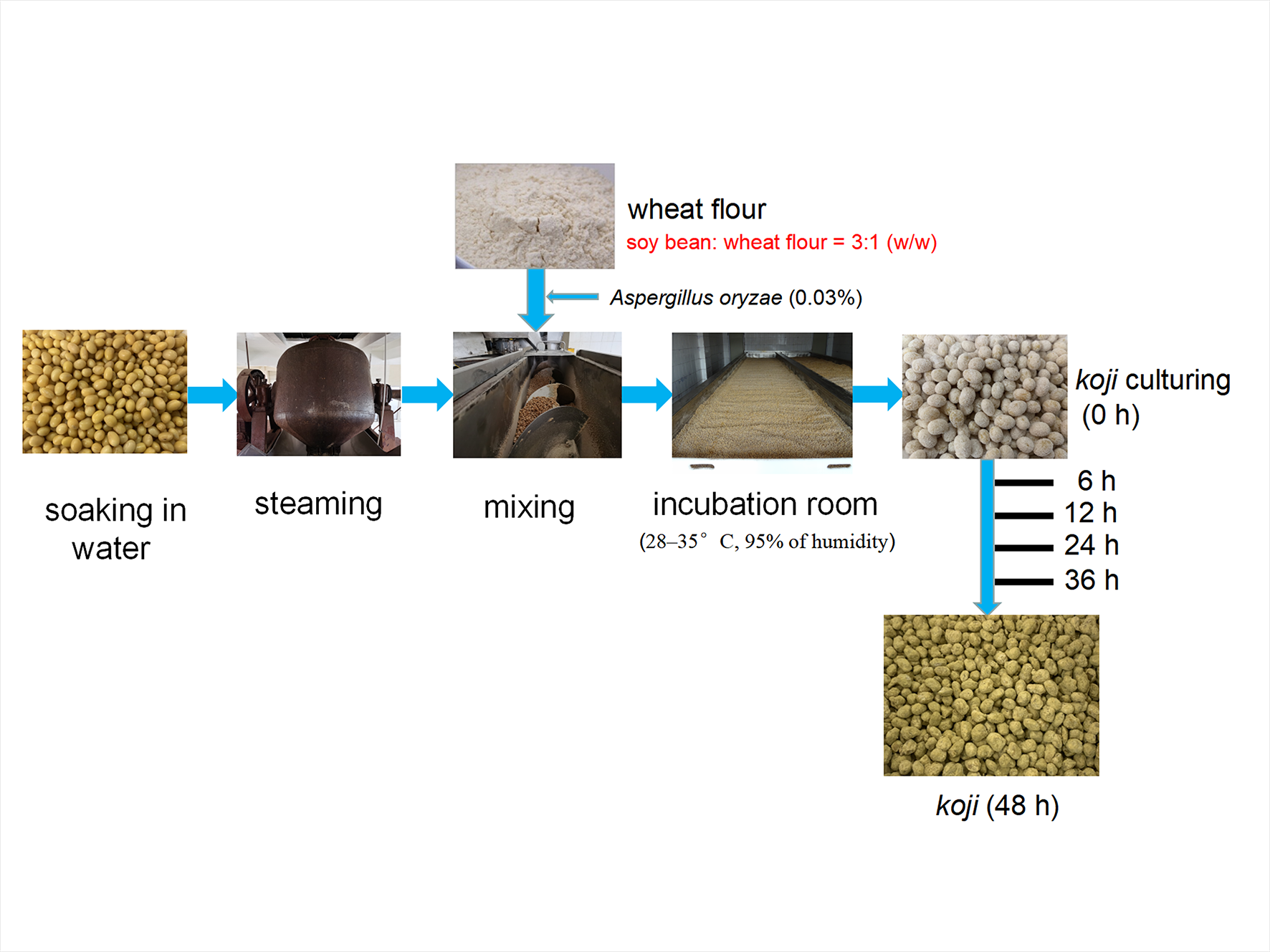

Supplement: Supplementary Figure 1 — Koji making overview and sampling times. [file Image_1.TIF]

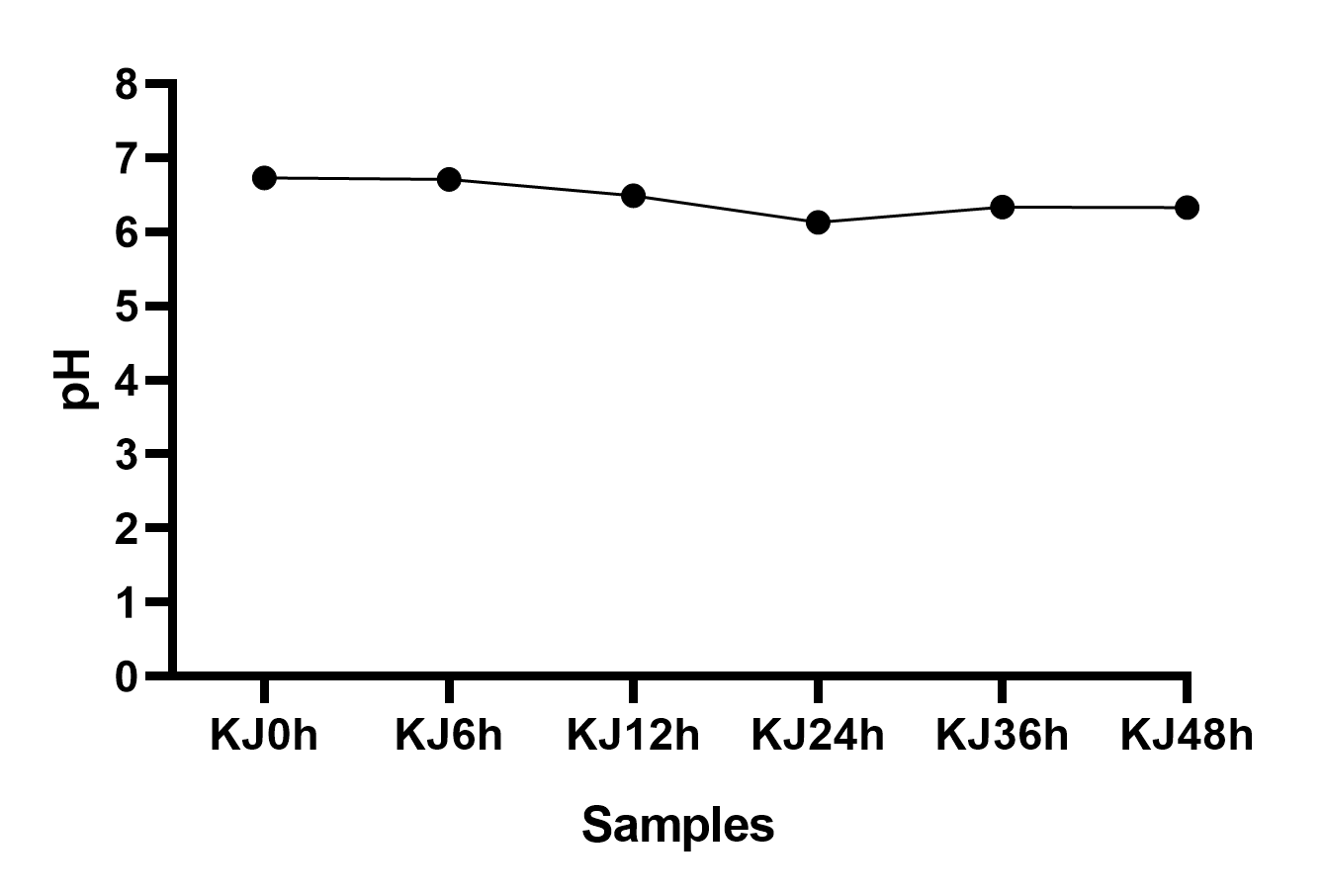

Supplement: Supplementary Figure 2 — pH changes during koji making. [file Image_2.TIF]

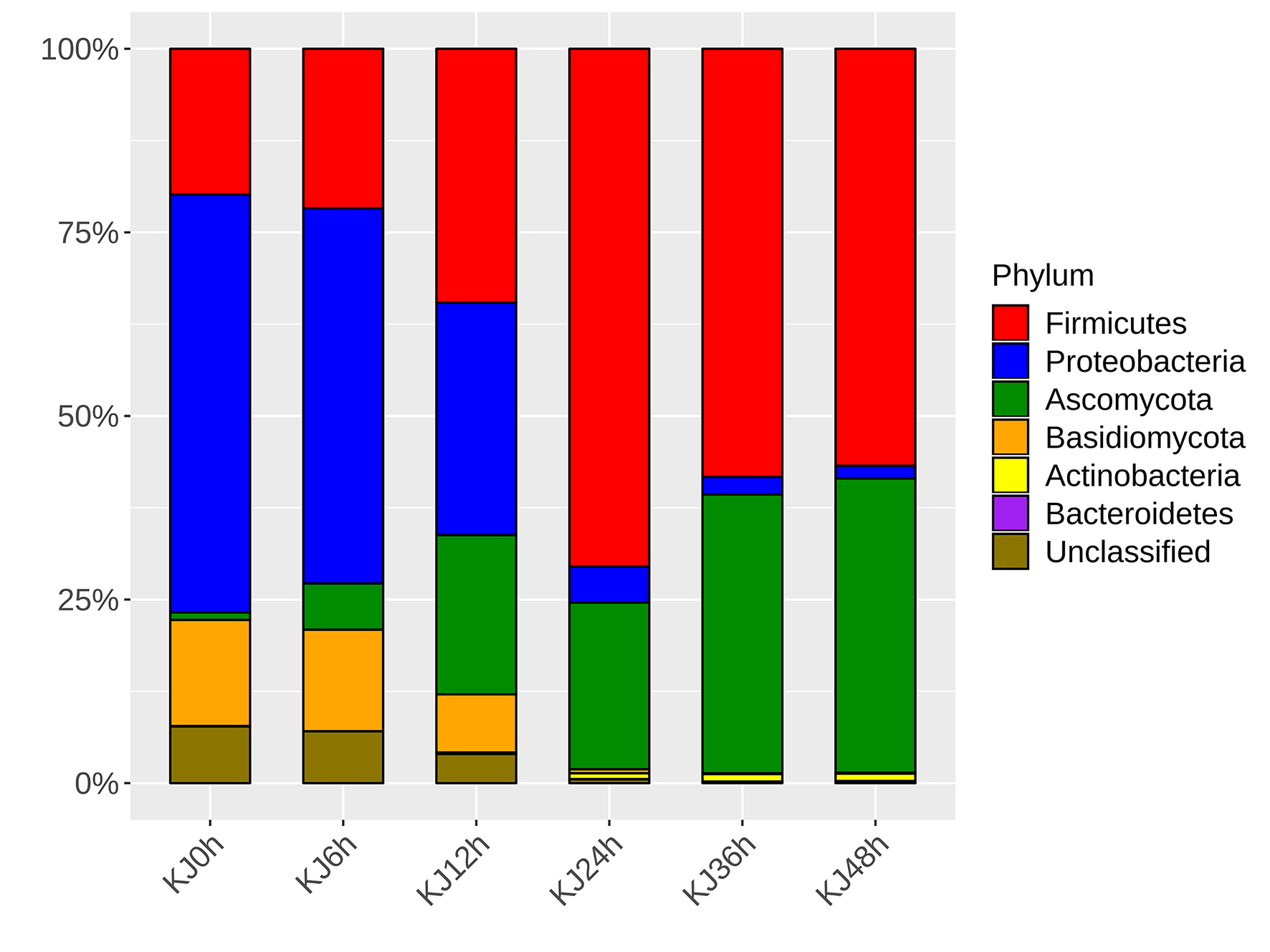

Supplement: Supplementary Figure 3 — Taxonomic composition of koji metagenomes at the phylum level showing changes in microbial communities across six fermentation stages (0–48 h). Sequences that could not be classified to known taxa were designated as “unclassified”. [file Image_3.TIF]

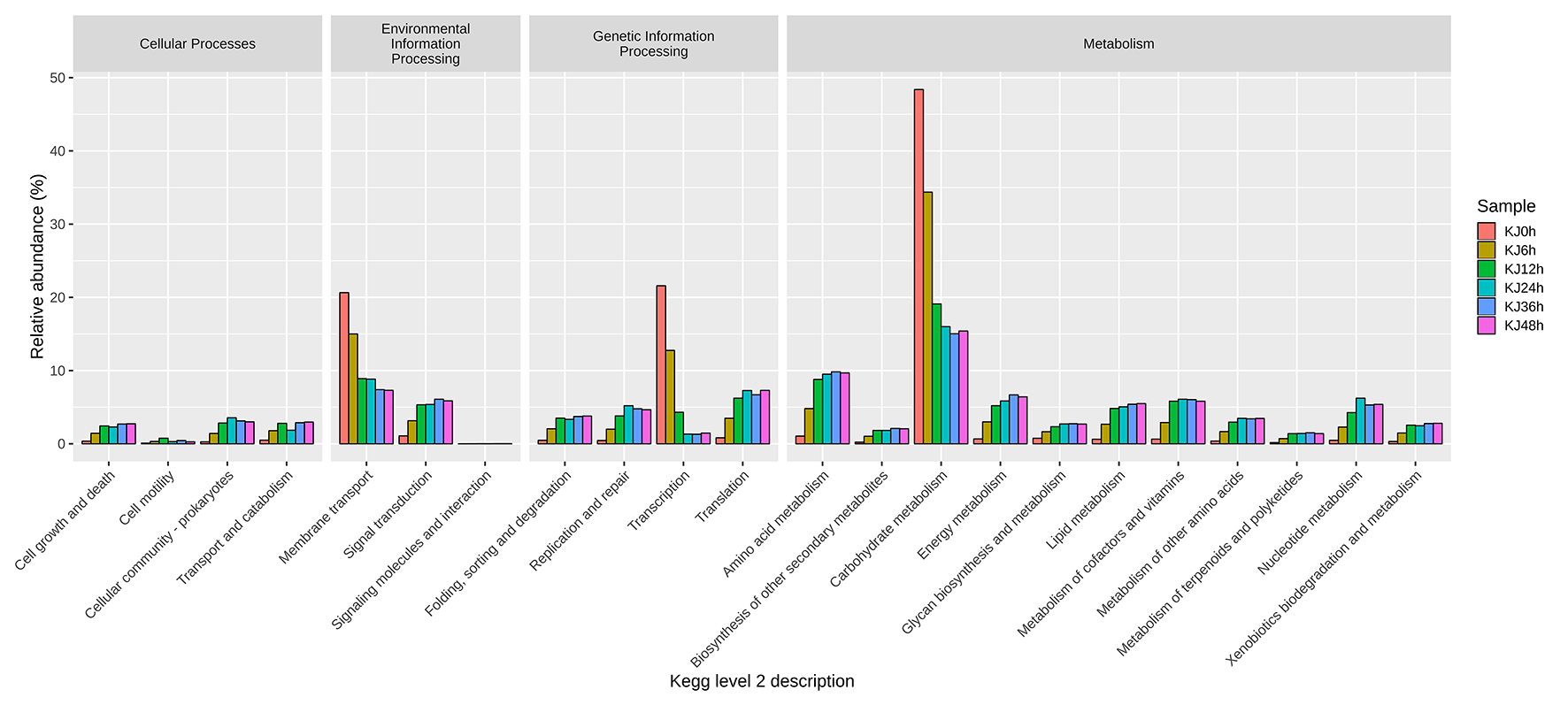

Supplement: Supplementary Figure 4 — Functional profiles produced from whole shotgun metagenome-derived ORFs annotated at level 2 of the Kyoto Encyclopedia of Genes and Genomes (KEGG) database. [file Image_4.TIF]
